# Supplementary figures and images for: Seascape genomics reveals limited dispersal and suggests spatially varying selection among European populations of sea lamprey (Petromyzon marinus)
Source: Evol Appl. 2023 May 27;16(6):1169–83. doi: 10.1111/eva.13561 (PMC10286227; doi:10.1111/eva.13561)

# Host community per area

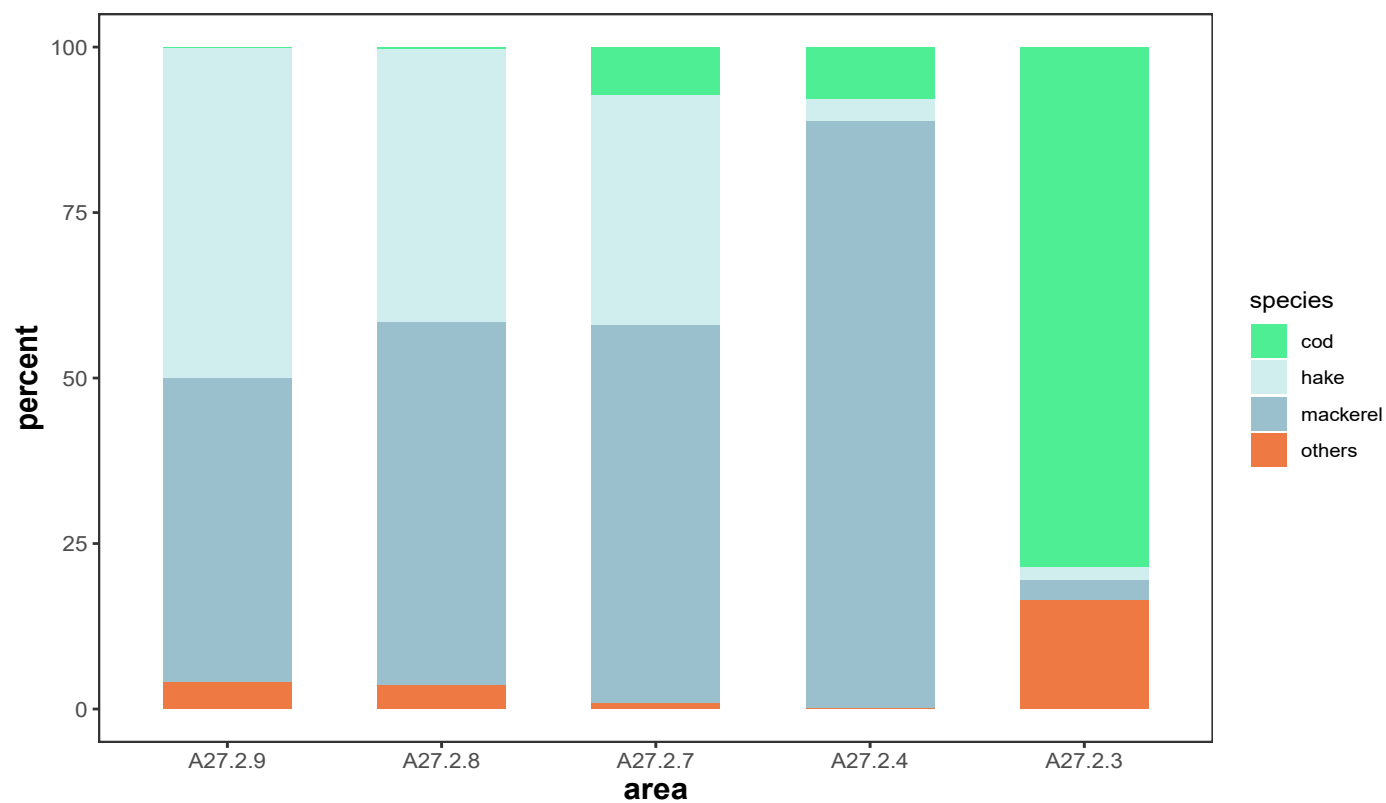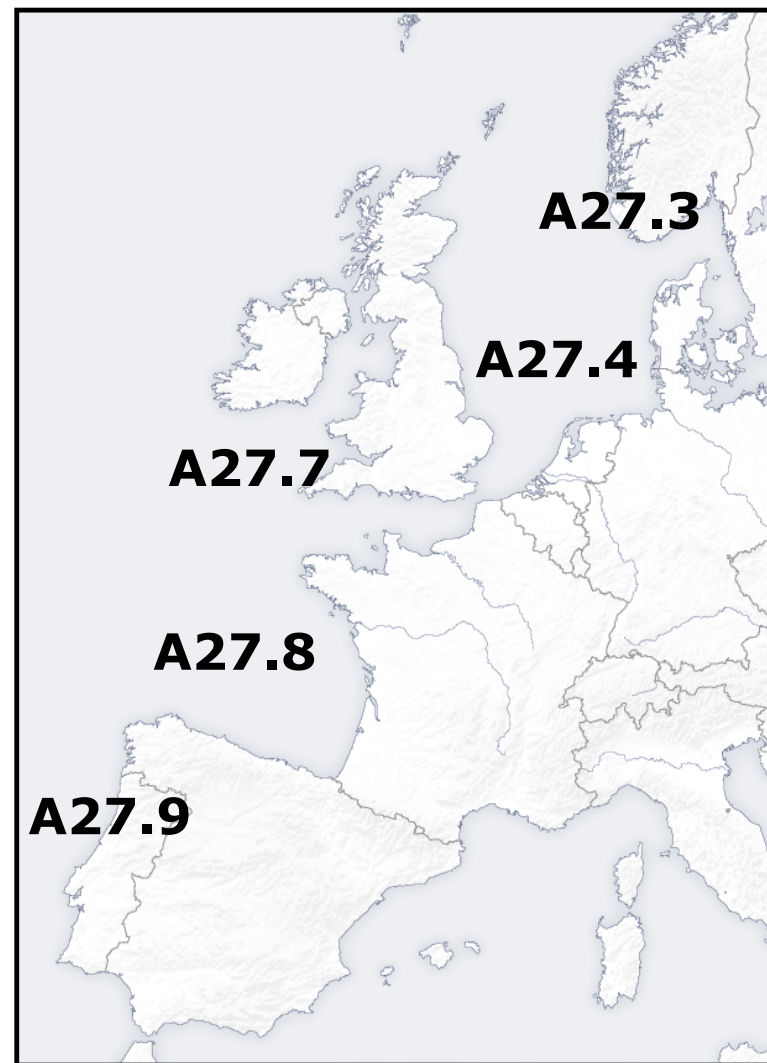

Supplement: Supplementary file 1 — Figure S1. [file EVA-16-1169-s010.pdf]

Projection onto PC1 and PC2

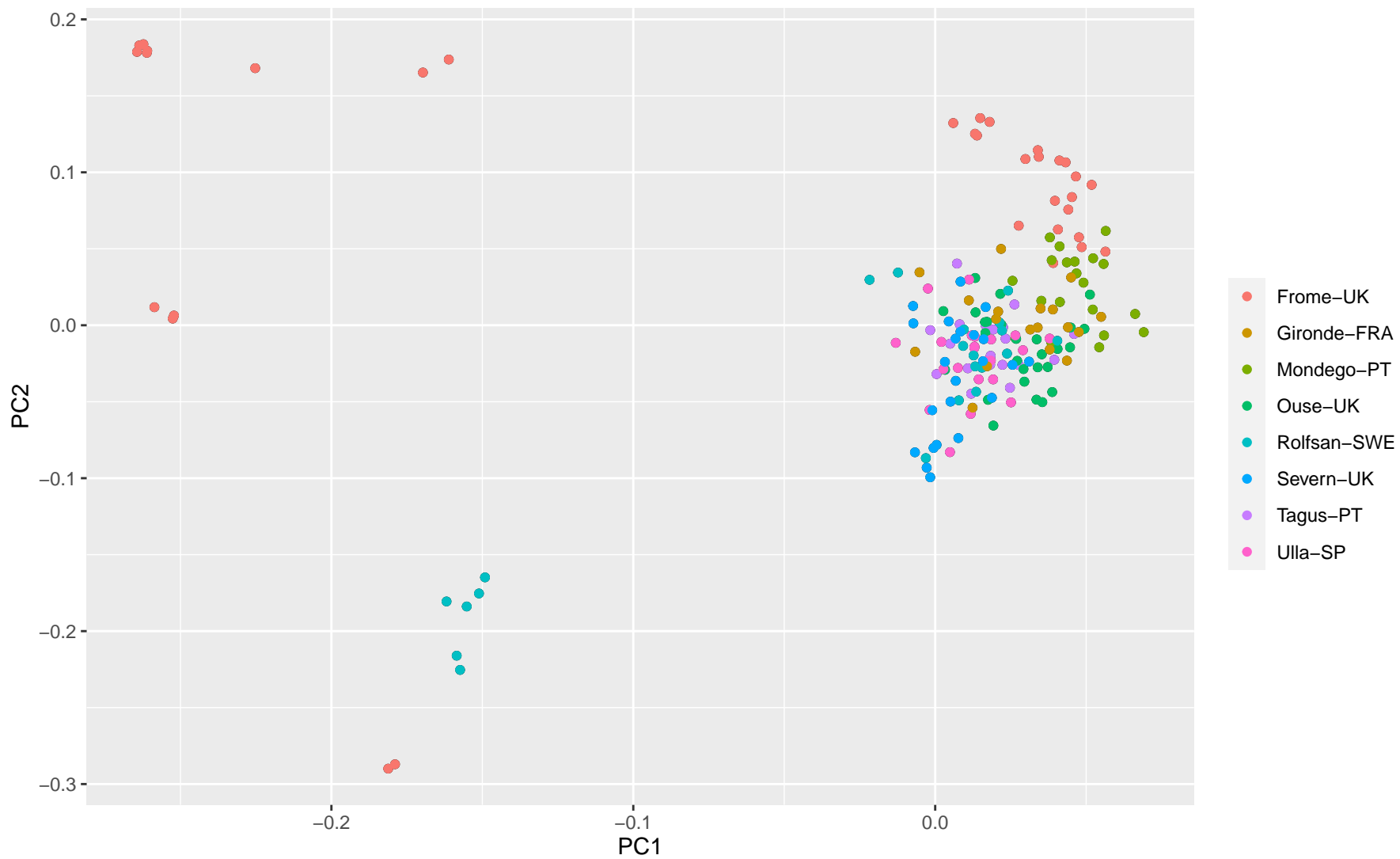

Supplement: Supplementary file 2 — Figure S2. [file EVA-16-1169-s006.pdf]

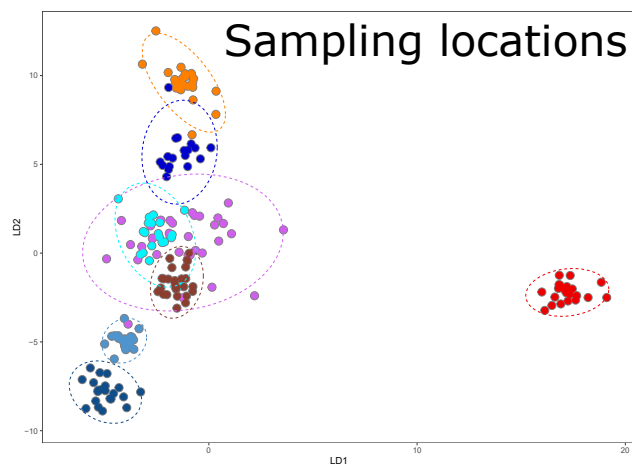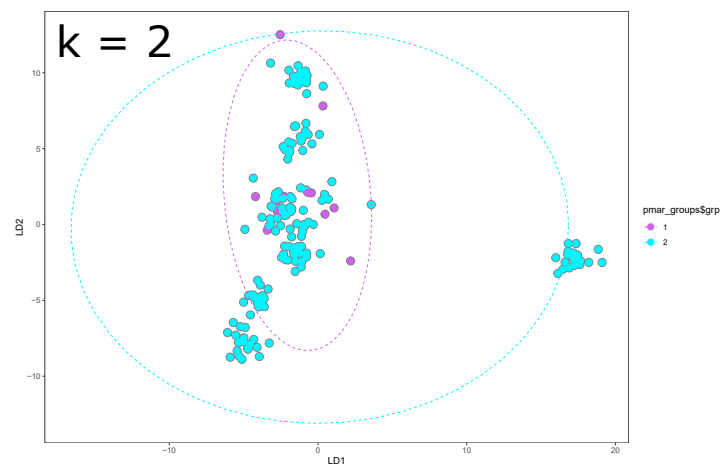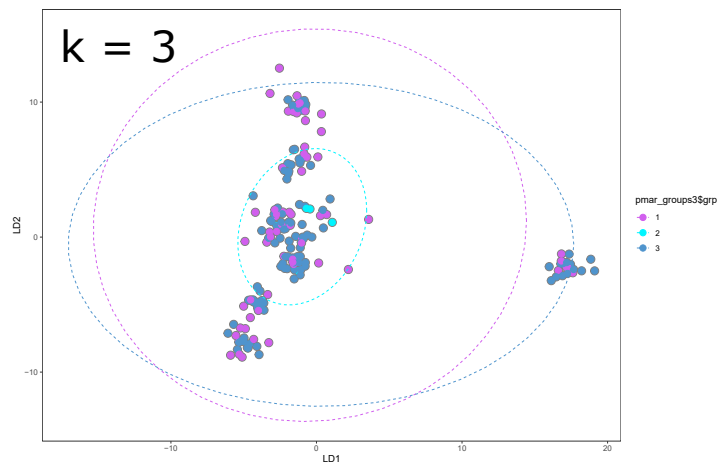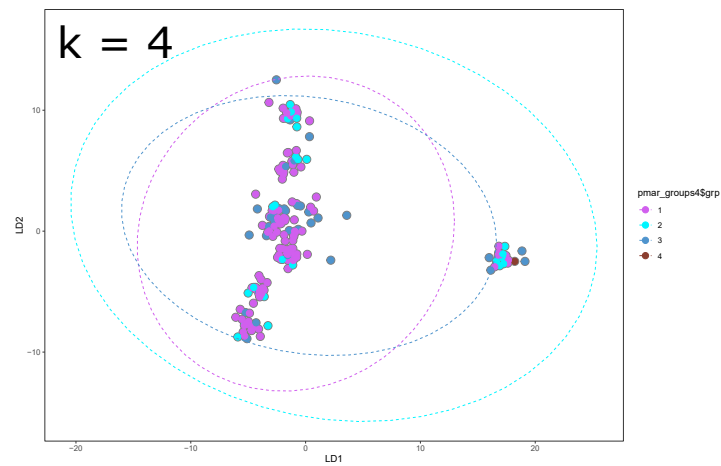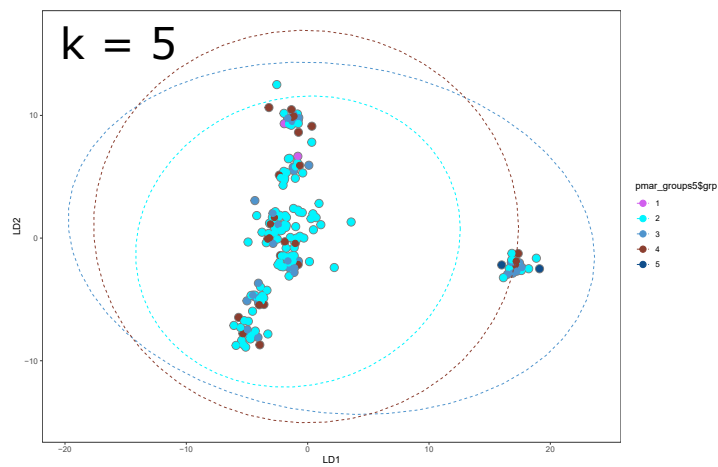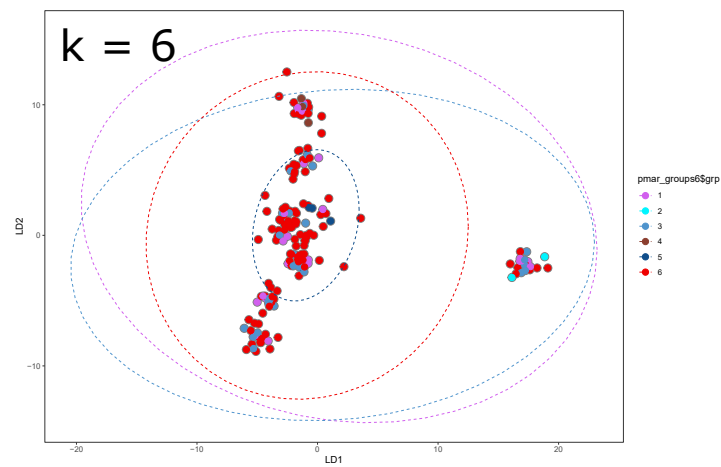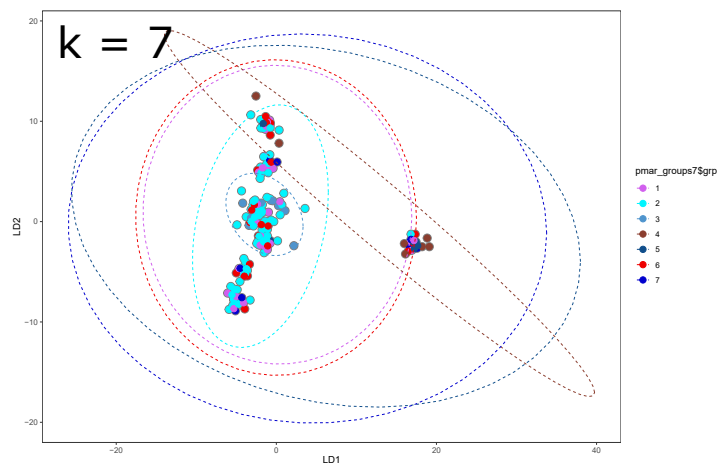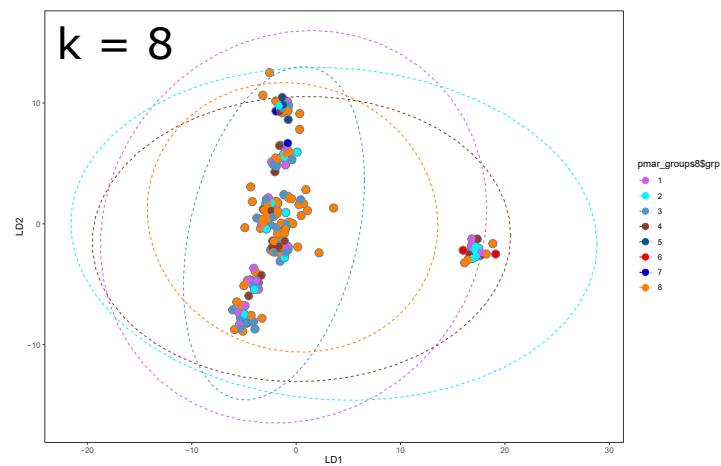

Supplement: Supplementary file 3 — Figure S3. [file EVA-16-1169-s008.pdf]

## a-score optimisation – spline interpolation

Optimal number of PCs: 65

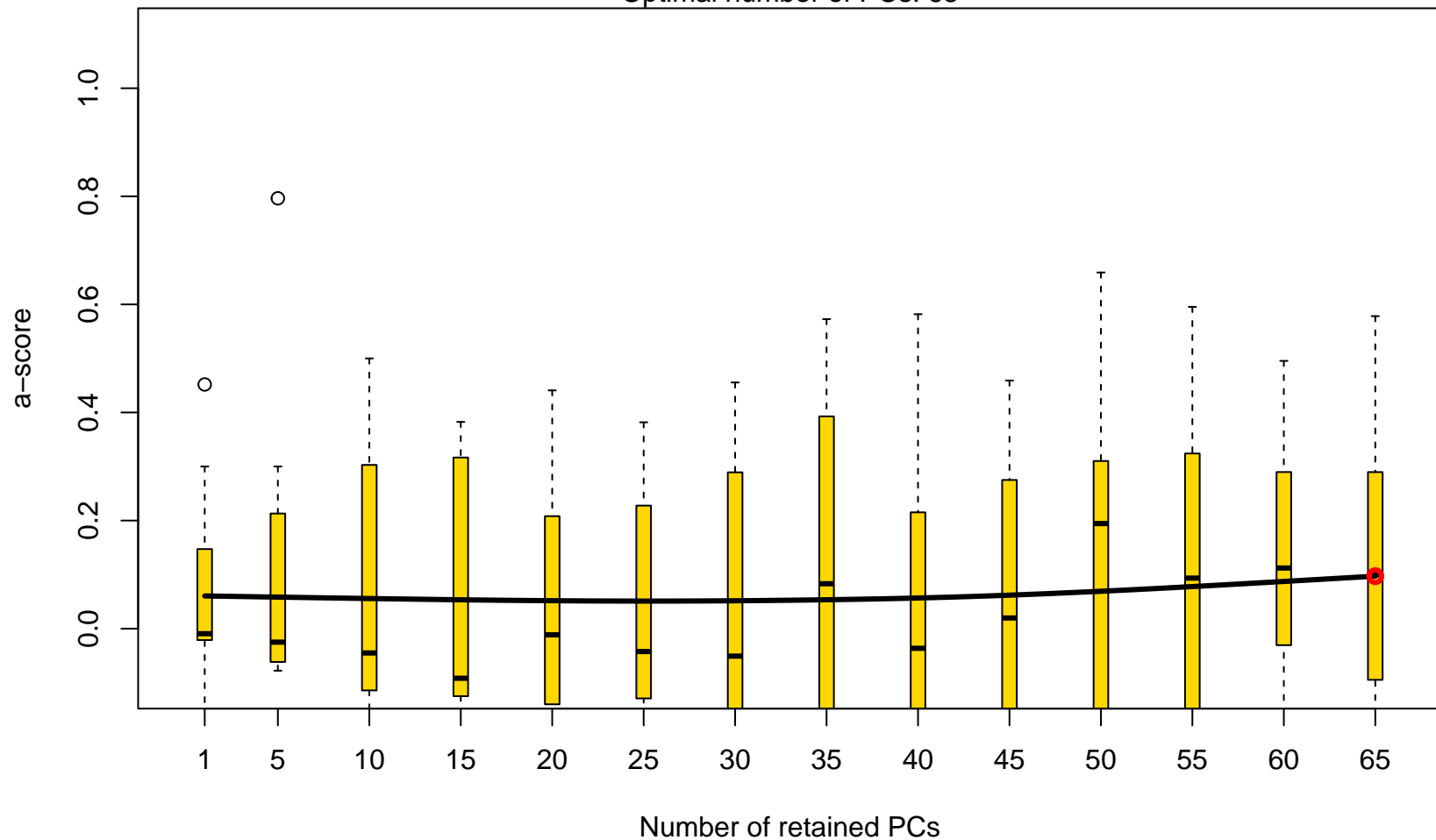

Supplement: Supplementary file 5 — Figure S5. [file EVA-16-1169-s001.pdf]
