# Supplementary material for: Seascape genomics reveals limited dispersal and suggests spatially varying selection among European populations of sea lamprey (Petromyzon marinus)
Source: Evol Appl. 2023 May 27;16(6):1169–83. doi: 10.1111/eva.13561 (PMC10286227; doi:10.1111/eva.13561)
Supplement: Supplementary file 4 — Figure S4. [file EVA-16-1169-s002.pdf]

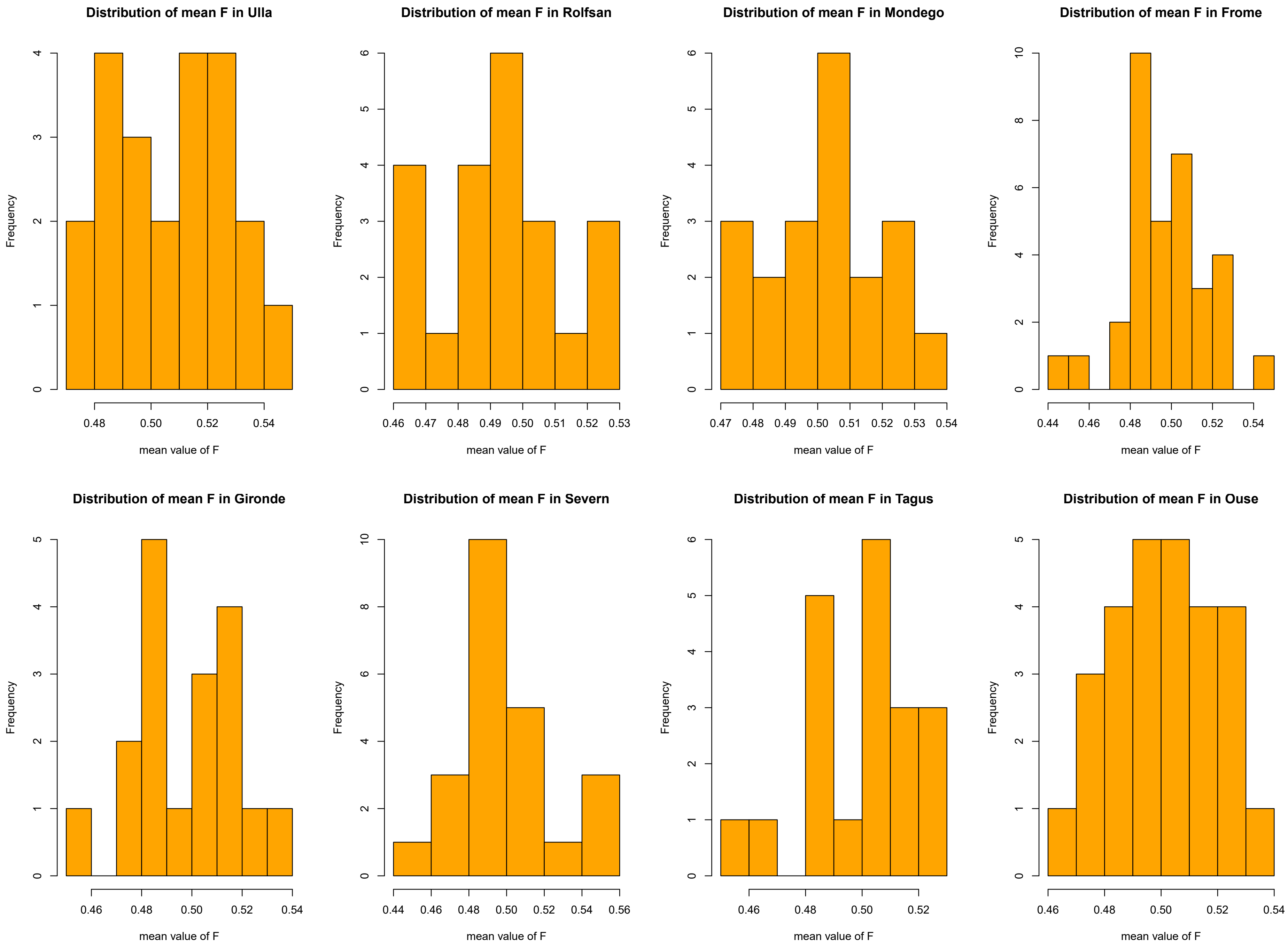
Distribution of mean F in Ulla
Distribution of mean F in Rolfsan
Distribution of mean F in Mondego
Distribution of mean F in Frome
Distribution of mean F in Gironde
Distribution of mean F in Severn
Distribution of mean F in Tagus
Distribution of mean F in Ouse
Frequency
mean value of F
